# The joint effects of physical activity and air pollution on type 2 diabetes in older adults

**DOI:** 10.1186/s12877-022-03139-8

**Published:** 2022-06-01

**Authors:** Linjun Ao, Junmin Zhou, Mingming Han, Hong Li, Yajie Li, Yongyue Pan, Jiayi Chen, Xiaofen Xie, Ye Jiang, Jing Wei, Gongbo Chen, Shanshan Li, Yuming Guo, Feng Hong, Zhifeng Li, Xiong Xiao, Xing Zhao

**Affiliations:** 1 West China School of Public Health and West China Fourth Hospital, Sichuan University, Chengdu Sichuan, China; 2 Chengdu Center for Disease Control and Prevention, Sichuan, China; 3 Yunnan Center for Disease Control and Prevention, Yunnan, China; 4 Tibet Center for Disease Control and Prevention CN, Tibet, China; 5 Tibet University, Tibet, China; 6 Department of Atmospheric and Oceanic Science, Earth System Science Interdisciplinary Center, University of Maryland, College Park, MD USA; 7 Guangdong Provincial Engineering Technology Research Center of Environmental and Health Risk Assessment, Department of Occupational and Environmental Health, School of Public Health, Sun Yat-sen University, Guangzhou, Guangdong China; 8 Department of Epidemiology and Preventive Medicine, School of Public Health and Preventive Medicine, Monash University, Melbourne, Australia; 9 School of Public Health, the key Laboratory of Environmental Pollution Monitoring and Disease Control, Ministry of Education, Guizhou Medical University, Guiyang, China; 10 Chongqing Municipal Center for Disease Control and Prevention, Chongqing, China

**Keywords:** Older adults, Physical activity, Type 2 diabetes, Air pollution, Joint effects

## Abstract

**Background:**

Older adults with type 2 diabetes are at higher risk of developing common geriatric syndromes and have a lower quality of life. To prevent type 2 diabetes in older adults, it’s unclear whether the health benefits of physical activity (PA) will be influenced by the harms caused by increased exposure to air pollution during PA, especially in developing countries with severe air pollution problem. We aimed to investigate the joint effects of PA and long-term exposure to air pollution on the type 2 diabetes in older adults from China.

**Methods:**

This cross-sectional study was based on the China Multi-Ethnic cohort (CMEC) study. The metabolic equivalent of PA was calculated according to the PA scale during the CMEC baseline survey. High resolution air pollution datasets (PM_10_, PM_2.5_ and PM_1_) were collected from open products. The joint effects were assessed by the marginal structural mean model with generalized propensity score.

**Results:**

A total of 36,562 participants aged 50 to 79 years were included in the study. The prevalence of type 2 diabetes was 10.88%. The mean (SD) level of PA was 24.93 (18.60) MET-h/d, and the mean (SD) level of PM_10_, PM_2.5_, and PM_1_ were 70.00 (23.32) µg/m^3^, 40.45 (15.66) µg/m^3^ and 27.62 (6.51) µg/m^3^, respectively. With PM_10_ < 92 µg/m^3^, PM_2.5_ < 61 µg/m^3^, and PM_1_ < 36 µg/m^3^, the benefit effects of PA on type 2 diabetes was significantly greater than the harms due to PMs when PA levels were roughly below 80 MET-h/d. With PM_10_ ≥ 92 µg/m^3^, PM_2.5_ ≥ 61 µg/m^3^, and PM_1_ ≥ 36 µg/m^3^, the odds ratio (OR) first decreased and then rose rapidly with confidence intervals progressively greater than 1 and break-even points close to or even below 40 MET-h/d.

**Conclusions:**

Our findings implied that for the prevention of type 2 diabetes in older adults, the PA health benefits outweighed the harms of air pollution except in extreme air pollution situations, and suggested that when the air quality of residence is severe, the PA levels should ideally not exceed 40 MET-h/d.

**Supplementary Information:**

The online version contains supplementary material available at 10.1186/s12877-022-03139-8.

## Introduction

Diabetes is a metabolic disorder caused by environmental and genetic factors and has been considered as one of the major contributors to the global burden of disease [1, 2]. Nearly 80% of people with diabetes, mainly type 2, now live in low- and middle-income countries [3, 4], and population aging have contributed to the shift of the diabetes epidemic to the elderly [5, 6]. Old adults with diabetes have a higher risk of common geriatric syndromes, such as cognitive impairment and disability, which have an important impact on quality of life [7].

Research has consistently demonstrated that the adoption of physical activity (PA) can prevent diabetes [8–10], especially among older adults [11, 12]. Long-term exposure to air pollution has been a critical risk factor for the development of diabetes [13–15], and older people would have a higher risk than younger ones [16, 17]. Given that PA increases the ventilation rate, the intake of air pollution may also increase. However, for older adults, the trade-off between the health benefits of PA and the harmful effects caused by increased exposure to air pollution during PA remains unclear [18].

Some studies have revealed the joint effects of PA and air pollution on cardiovascular disease, lung function/respiratory disease and mortality [19–28], but evidence on the joint effects of long-term exposure to air pollution and PA on type 2 diabetes is scarce [28–30]. Besides, no such evidence existed in developing countries, which faced much more serious air pollution problems [31]. As the largest developing country in the world, China has widespread and severe levels of air pollution, with 48 cities feature among the top 100 most polluted cities, and previous studies conducted with good air quality could not be extrapolated to such a high-exposure air pollution settings [27, 32].

We aimed to investigate the joint effects of PA and long-term exposure to air pollution on type 2 diabetes in older adults exposed to heavy air pollution from China, where air pollution and the prevalence of type 2 diabetes both pose a grave public health concern [4, 33].

## Methods

### Study design and participants

This cross-sectional study was based on the China Multi-Ethnic cohort (CMEC), documented in detail elsewhere [34]. In brief, the baseline for the CMEC study was established between May 2018 and September 2019 in five provinces in southwest China, which sampled 99,556 participants aged 30 to 79 years. Electronic questionnaires, physical examinations and clinical laboratory tests were mainly applied to collect participants’ baseline information, such as demographic and socioeconomic information, health behaviours, disease history, and biological samples.

The participant selection procedure is shown in the Data Supplement (Fig S1). Tibetans in Aba and Lhasa live above 3000 m above sea level. High altitude has been documented to be inversely associated with diabetes due to adaptation to environments and genetic changes [35, 36]. Besides, Tibetans in Aba were herdsmen whose residence changed with the seasons. Thus, to make the study population more comparable and obtain accurate and stable exposure estimates, Tibetan residents in Aba and Lhasa were not included in this study (*n* = 4993 for Aba; *n* = 7780 for Lasa). We then excluded 9372 participants who had changed their place of residence within three years prior to the baseline survey, and excluded 2739 participants diagnosed with type 2 diabetes before exposure assessment. We further excluded 672 participants who self-reported having cancers, mainly lung, oesophageal, stomach, liver, prostate and cervical cancers, as well as 2775 pregnant women and 161 participants self-reported having tuberculosis. We then selected adults over 50 years of age and excluded 1237 participants due to incomplete information on air pollution exposure, PA, health outcomes and other covariates. A total of 36,562 adults aged 50 to 79 years were included in this study.

### Assessment of air pollution exposure

The high-resolution (1 km) and high-quality PM_10_ (particulate matter with an aerodynamic diameter of 10 µm or less), PM_2.5_ (particulate matter with an aerodynamic diameter of 2.5 µm or less) and PM_1_ (particulate matter with an aerodynamic diameter of 1 µm or less) dataset were collected from open products, which were estimated using a newly developed space–time extremely randomized trees (STET) model based on the newly released MODIS Collection 6 MAIAC 1-km AOD products, meteorological variables, pollution emissions, land cover, surface topographic data and population data [37–40]. The STET model performed well, with an across-validation coefficient of determination of 0.86, 0.90, and 0.77 for PM_10_, PM_2.5_ and PM_1_, respectively.

**Fig. 1:**
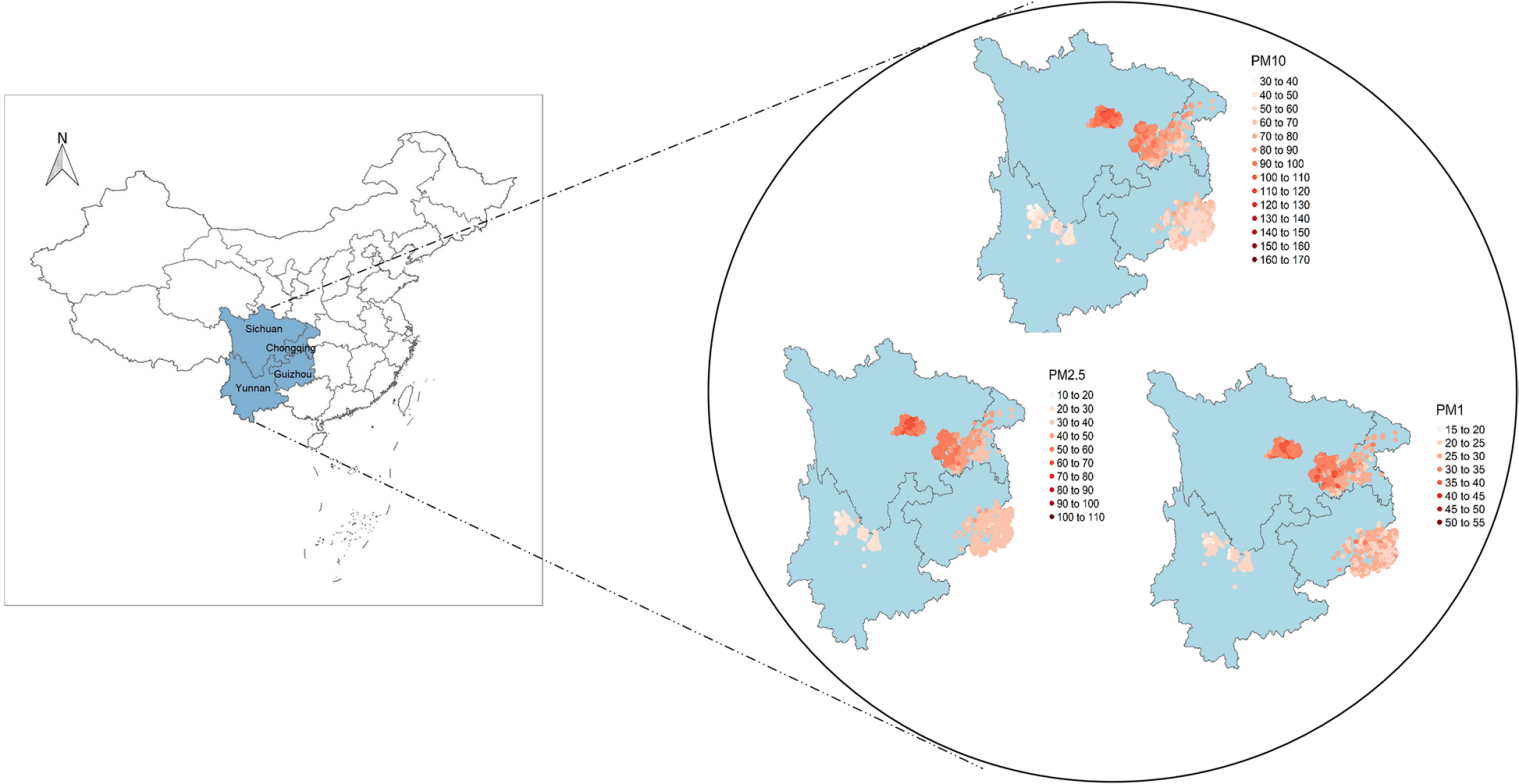
The geographical distribution of PM_10_, PM_2.5_ and PM_1_ concentrations by participants’ address locations

We assigned the estimated annual PM_10_, PM_2.5_ and PM_1_ concentrations to each participant based on their geocoded residential address and calculated the 3-year average exposure concentrations before the baseline survey. Figure 1 showed the distribution of PM_10_, PM_2.5_ and PM_1_ concentrations by participants’ address locations.

### Assessment of physical activity

The information of PA for each participant was collected through the questionnaire during the baseline survey. Both the PA intensity and duration one year preceding the survey were obtained. The PA intensity was presented by the corresponding metabolic equivalent values (MET). This study assigned different MET to various physical activities [41–43], and the product of PA intensity (MET) and duration (hours) was calculated as the volume of activity (MET-h/d). Measures of PA were calculated corresponding to four domains, namely, leisure, work, transportation and housework. The sum of activity in each domain were the total volume of PA for each participant.

### Health outcome measurements

The diabetes status of the participants was defined by the following criteria: (1) self-reporting of taking any antidiabetic medication (both insulin and oral antidiabetic drugs) or (2) fasting plasma glucose ≥ 126 mg/dL (7.0 mmol/L), or (3) HbA_1c_ ≥ 6.5% (48 mmol/mol). Those criteria were based on the recommendations of the American Diabetes Association [44].

### Covariates

In this study, we included the following covariates in the main analysis: age (year), sex (male or female), marital status (married, widowed, divorced and unmarried), education (illiteracy, primary school degree, junior school degree, senior high school degree, bachelor’s degree or more), annual household income, body mass index (low, normal, overweight and obese), smoking status (never, quit, smoking), passive smoking status (yes or no), alternative Mediterranean diet (aMED) score [45], self-reported hypertension (yes or no), sedentary time, and indoor pollution situation. Indoor pollution was defined as low if participants did not have a kitchen at home or rarely cooked, medium if they frequently cooked using gas/electricity or using coal/wood as fuel with chimney at home, and high if they frequently cooked using coal/wood as fuel but without a chimney at home [46].

### Statistical analysis

#### Basic theory of the bi-dimensional GPS

Causal inference methods are gradually being used in environmental epidemiology because of better control of confounding, and avoiding ethical issues [47, 48]. Among various causal inference methods, propensity score methods have been popular for its advantage of separating the design and analysis process, which is similar to randomized clinical trials [49]. Based on the propensity score, the inverse probability of treatment weighting (IPTW) method has been widely used due to its ease of operation and unbiased estimation [50]. The IPTW method currently focuses on categorical or single continuous exposure variables, while research on two continuous variables is lacking.

In this study, let $T$ and $V$ denote PA and air pollution, respectively, and $X$ denote covariates measured in the baseline survey described above. Based on the weak unconfoundedness assumption and the generalised propensity score (GPS) proposed by Hirano and Imbens [51], we extended the weak unconfoundedness assumption, namely $Y\left(t,v\right)\perp (T,V)|X$, and developed a bi-dimensional GPS by as follows:

$$R=r\left(T,V,X\right)$$

$$r\left(t,v,x\right)={f}_{T,V|X}\left(t,v|x\right).$$

Let $r\left(t,v,x\right)$ denote the conditional joint density of the two continuous exposure variables given the covariates. Similar to the GPS [51], the bi-dimensional GPS also has a balancing property as follows:

$$X\perp 1\left\{T=t,V=v\right\}|r\left(t,v,X\right)$$

The above balancing property implies that given the bi-dimensional GPS, covariates $X$ are balanced across different joint exposure groups and will not interfere with the estimation of the association between exposure and outcome variables. This is a mechanical implication of the definition of the bi-dimensional GPS, and does not require the extended weak unconfoundedness assumption. Combined with the extended weak unconfoundedness, this implies that assignment to treatment is unconfounded given the bi-dimensional GPS (proof in supplementary S1):

$${f}_{T,V}\left\{\left(t,v\right)|r\left(t,v,X\right),Y\left(t,v\right)\right\}={f}_{T,V}\left\{\left(t,v\right)|r\left(t,v,X\right)\right\}$$

#### Design stage

The overall workflow of the IPTW method was presented in supplementary (Fig S2), and the estimation of bi-dimensional GPS was the first step. Due to a lack of research about the application of IPTW on two continuous variables, we proposed to construct a bi-dimensional GPS by using the multivariate normal model:

$$\left(\genfrac{}{}{0pt}{}{E\left(T\right)}{E\left(V\right)}\right)=\left(\genfrac{}{}{0pt}{}{{\beta }_{01}+{X}_{1}{\beta }_{11}+{X}_{2}{\beta }_{21}+{X}_{3}{\beta }_{31}+\dots +{X}_{p}{\beta }_{p1}}{{\beta }_{02}+{X}_{1}{\beta }_{12}+{X}_{2}{\beta }_{22}+{X}_{3}{\beta }_{32}+\dots +{X}_{p}{\beta }_{p2}}\right)$$

${X}_{1},{X}_{2},\dots ,{X}_{p}$ were the relevant covariates mentioned above, and the subscript $p$ represented the number of covariates parameters. A weighted pseudo population was created by the bi-dimensional GPS. The evaluation of covariate balance in the pseudo population is a crucial step in the causal inference framework, which indicates the quality of the causal inference approach at recovering randomized experiments and informs the degree to which we can make a valid causal assessment. The balance was measured through the absolute correlation (AC) between the continuous exposure variables and the covariates. The AC with values < 0.1 indicates a high quality in recovering randomized experiments [52].

#### Outcome analysis stage

A marginal structural mean model [53] was constructed to assess the joint effects of PA and long-term exposure to air pollution (PM_10_, PM_2.5_ and PM_1_) on type 2 diabetes, which combined the bi-dimensional GPS and the generalized additive model [54]. The model was specified as:

$$E\left[{Y}^{t,v}\right]={\beta }_{0}+Te\left(t*v\right)$$

$$={\beta }_{0}+Ti\left(t\right)+Ti\left(v\right)+Ti\left(t,v\right)$$

The variable $Te$ defined the full tensor product smooth between PA and long-term exposure to air pollution, and the right-hand part of the second equal was a functional ANOVA decomposition with the smooth main effects ($Ti\left(t\right)+Ti\left(v\right)$) and smooth interaction term ($Ti\left(t,v\right)$). F-statistic was used for the test of the smooth interaction term [54].

To present the results clearly without using 3D plots, we investigated the exposure–response relationship between PA and type 2 diabetes at different concentrations of PMs. Participants exposed to the lowest PMs and lowest PA levels were the reference group. The choice of different PMs concentrations was based on its distribution and the WHO recommendations. Specifically, the nine levels of PM_10_ were 40 50, 65, 70, 88, 92, 104, 107, 120 µg/m^3^; the nine levels of PM_2.5_ were 20, 25, 35, 48, 52, 54, 61, 65, 70 µg/m^3^; and the nine levels of PM_1_ were 18, 21, 23, 26, 32, 33, 36, 38 and 42 µg/m^3^.

Sensitivity analyses were performed to assess the robustness of joint effects: (1) the above main analyses were repeated after excluding subjects taking any antidiabetic medication because their intentional lifestyle changes may produce estimate bias, such as doing more exercise to prevent exacerbation of the disease; (2) the 2-year average exposure of air pollution and 4-year average exposure of air pollution were employed to evaluate the possible impact of different exposure windows.

All statistical analysis were performed in R software, version 3.4.0.

## Results

### Descriptive results

A total of 36,562 participants were included in the study, and the prevalence of type 2 diabetes was 10.88%. Table 1 shows the characteristics of all participants, the participants without diabetes, and the participants with type 2 diabetes. For all participants, the average age was 60.54 (7.50) years old, and 58.9% were females. Compared to participants without type 2 diabetes, those with type 2 diabetes had lower levels of PA (MET-h/d) (22.65 *vs*. 25.21), and higher levels of long-term exposure to pollutants at their residence.

**Table 1:** The characteristics of the study participants

	Total	Individuals without type 2 diabetes	Individuals with type 2 diabetes	*p*
Number of people	36,562	32,584	3978	
Age, mean (SD)	60.54 (7.50)	60.38 (7.48)	61.86 (7.55)	< 0.001
Gender, n (%)				< 0.001
male	15,041 (41.1)	13,149 (40.4)	1892 (47.6)	
female	21,521 (58.9)	19,435 (59.6)	2086 (52.4)	
Marital status, n (%)				< 0.05
Married or cohabiting	31,446 (86.0)	28,064 (86.1)	3382 (85.0)	
Widowed	1095 (3.0)	992 (3.0)	103 (2.6)	
Separated or divorced	3908 (10.7)	3427 (10.5)	481 (12.1)	
Never married	113 (0.3)	101 (0.3)	12 (0.3)	
Income, n (%)				0.255
< 12,000 ¥	8648 (23.7)	7713 (23.7)	935 (23.5)	
12,000–19,999 ¥	7027 (19.2)	6280 (19.3)	747 (18.8)	
20,000–59,999 ¥	12,630 (34.5)	11,285 (34.6)	1345 (33.8)	
60,000–99,999 ¥	4815 (13.2)	4272 (13.1)	543 (13.7)	
> = 100 K ¥	3442 (9.4)	3034 (9.3)	408 (10.3)	
Education, n (%)				0.735
illiteracy	12,217 (33.4)	10,878 (33.4)	1339 (33.7)	
primary school	10,284 (28.1)	9196 (28.2)	1088 (27.4)	
junior high school	8649 (23.7)	7704 (23.6)	945 (23.8)	
senior high school	3818 (10.4)	3384 (10.4)	434 (10.9)	
bachelor or above	1594 (4.4)	1422 (4.4)	172 (4.3)	
BMI, mean (SD)	23.97 (3.43)	23.80 (3.36)	25.39 (3.64)	< 0.001
PA, mean (SD)	24.93 (18.60)	25.21 (18.64)	22.65 (18.09)	< 0.001
Smoking status, n (%)				< 0.001
never	26,259 (71.8)	23,604 (72.4)	2655 (66.7)	
quit	2384 (6.5)	2053 (6.3)	331 (8.3)	
smoke	7919 (21.7)	6927 (21.3)	992 (24.9)	
Second-hand smoke, n (%): yes	17,904 (49.0)	15,992 (49.1)	1912 (48.1)	0.233
Alcohol, n (%)				< 0.001
Never	21,851 (59.8)	19,505 (59.9)	2346 (59.0)	
Occasionally	8827 (24.1)	7921 (24.3)	906 (22.8)	
Often	5884 (16.1)	5158 (15.8)	726 (18.3)	
aMED score, mean (SD)	24.49 (4.51)	24.52 (4.50)	24.28 (4.56)	< 0.005
PM_10_ (µg/m^3^), mean (SD)	70.00 (23.32)	69.79 (23.29)	71.73 (23.56)	< 0.001
PM_2.5_ (µg/m^3^), mean (SD)	40.45 (15.66)	40.32 (15.64)	41.58 (15.73)	< 0.001
PM_1_ (µg/m^3^), mean (SD)	27.62 (6.51)	27.57 (6.51)	28.04 (6.48)	< 0.001

*PA* Physical activity, *BMI* Body mass index, *aMED* Alternative Mediterranean diet (aMED) score, *SD* Standard deviation

The exposure ranges of PM_10_, PM_2.5_ and PM_1_ were indeed wide, ranging from 33.26 µg/m^3^ to 165.19 µg/m^3^, 18.24 µg/m^3^ to 105.29 µg/m^3^ and 15.49 µg/m^3^ to 53.57 µg/m^3^, respectively (Fig. 1). Table S1 showed that the participants with higher PA tended to have lower prevalence of type 2 diabetes and be exposed to lower levels of PM_10_, PM_2.5_ and PM_1_.

### Balance check results

**Fig. 2:**
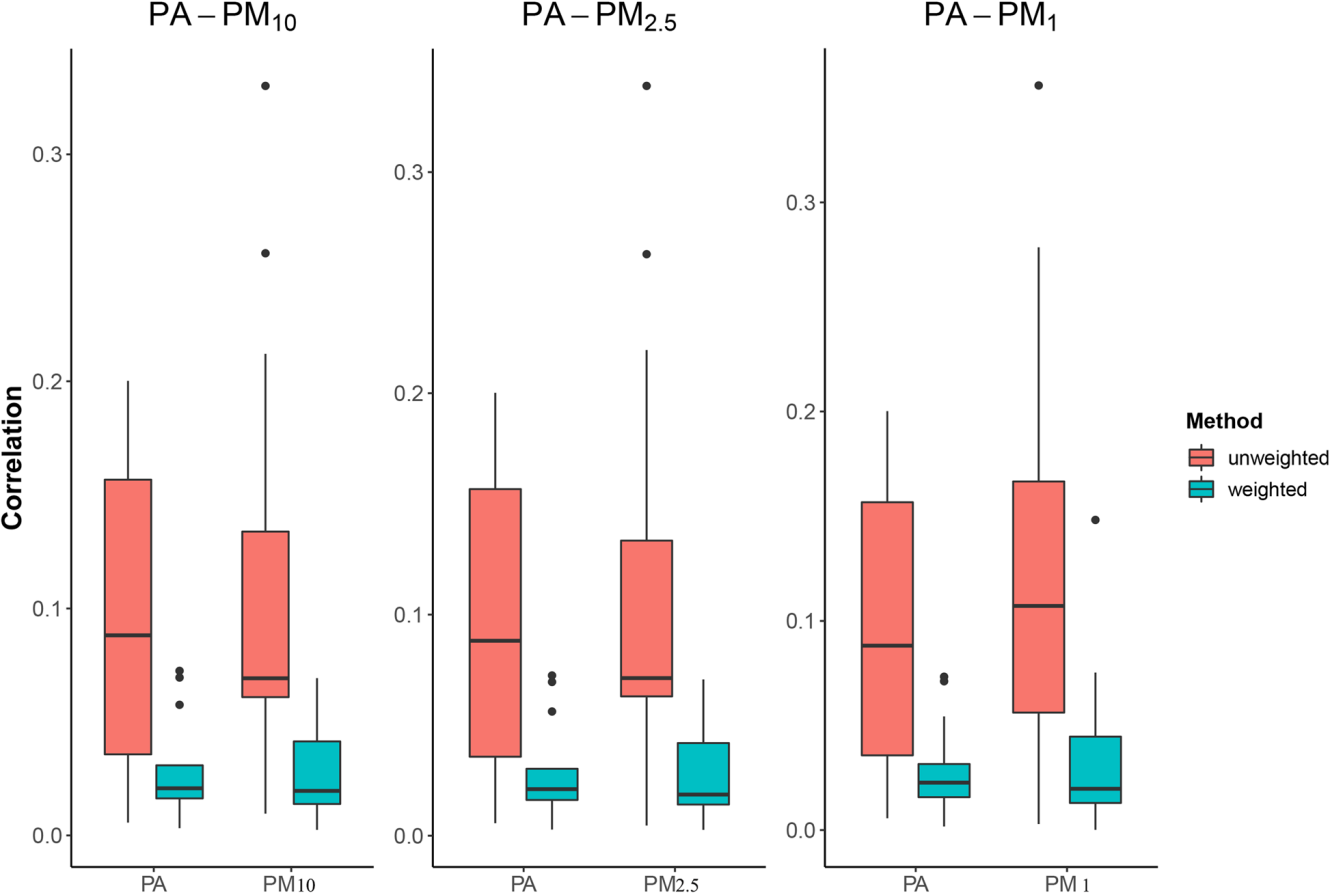
The balance results of covariates in the weighted (blue), and original observational population (red). The three subplots showed the ACs between covariates and PA and the corresponding pollutants before and after weighting with the bi-dimensional GPS model constructed for PA and PM_10_, PA and PM_2.5_, and PA and PM_1_, respectively. Covariates were age, sex, marital status, education, annual household income, BMI, smoking, aMED score, sedentary time, etc., as detailed in the Methods

As shown in Fig. 2, we found that compared with the unweighted study population, the covariates balance became better in the weighted pseudo-population, with values of ACs less than 0.1.

### The joint associations of PA and PMs on type 2 diabetes

There was a statistically significant interaction effect of PA and PMs on type 2 diabetes. The *p*-values for the smooth interaction term between PM_10_, PM_2.5_, and PM_1_ with PA in the generalised additive model were 4.73e-05, 3.00e-03, and 1.41e-07, respectively.

**Fig. 3:**
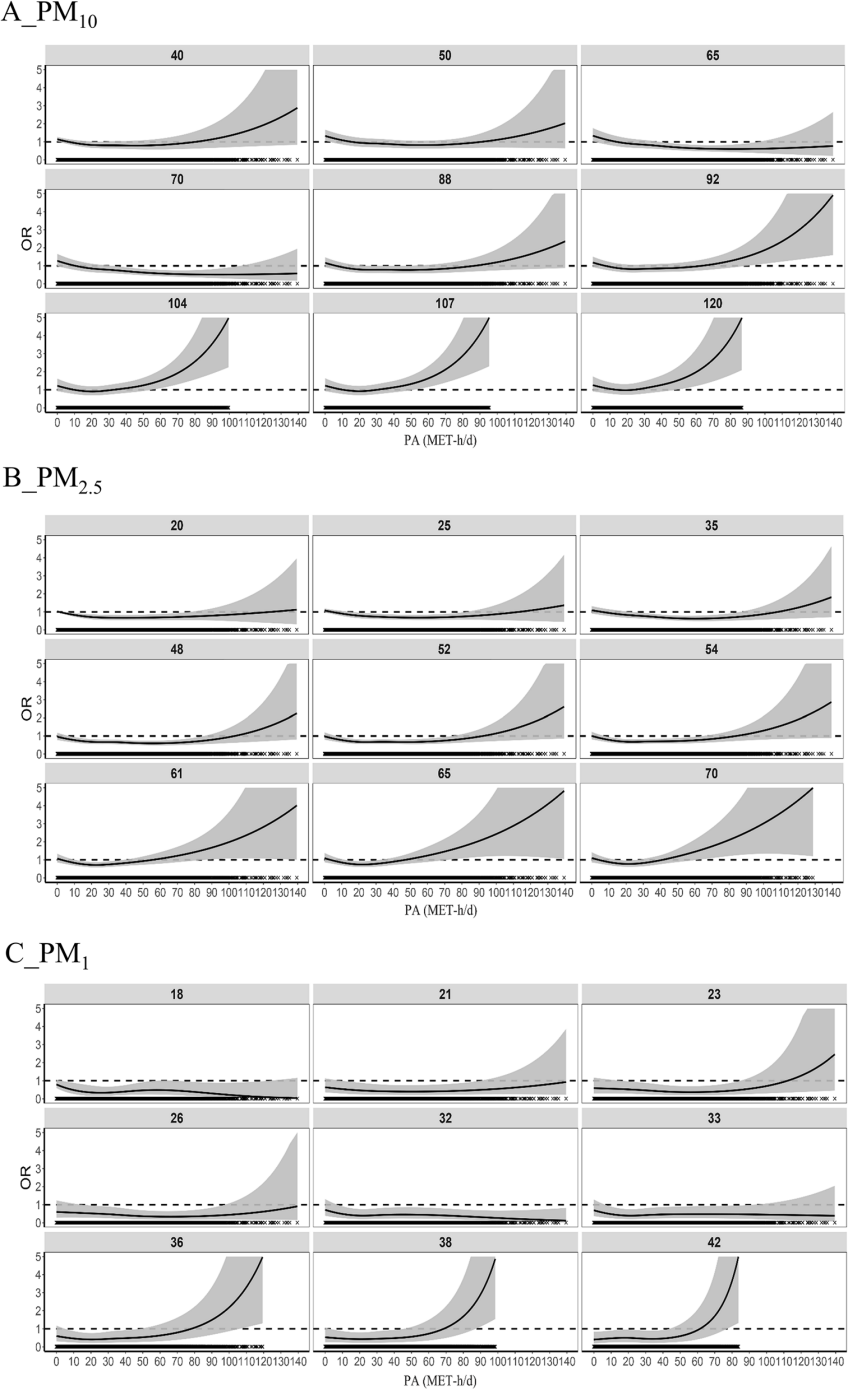
The exposure–response relationship between PA and type 2 diabetes at different exposure levels of air pollution for older adults. The nine values in subplots **A**, **B** and **C** represented the different pollution concentrations (µg/m^3^) of PM_10_, PM_2.5_ and PM_1_ respectively. The OR limit is set to 5, and the grey shaded area indicated the confidence interval (95% CI). The 95% CI not containing the value of 1, represented by the horizontal dashed line, indicated that the association is statistically significant. Covariates mentioned in the Methods section were integrated by the bi-dimensional GPS, which was combined in the outcome model

With PM_10_ < 92 µg/m^3^, PM_2.5_ < 61 µg/m^3^, and PM_1_ < 36 µg/m^3^, the exposure–response relationship between PA and type 2 diabetes showed that the OR first decreased, then remained stable and finally increased with increasing PA (Fig. 3). Overall, as the air pollution level increased, the break-even points, where the harmful effects from air pollution started to outweigh the benefits of physical activity, were roughly close to 80 MET-h/d. Specifically, the break-even points were 87 MET-h/d and 84 MET-h/d when PM_10_ was 88 µg/m^3^ and PM_2.5_ was 54 µg/m^3^, respectively.

With high levels of air pollution exposure, i.e. PM_10_ ≥ 92 µg/m^3^, PM_2.5_ ≥ 61 µg/m^3^, and PM_1_ ≥ 36 µg/m^3^, the exposure–response relationship showed that the OR first decreased and then rose rapidly from below 1 to above 1 with confidence intervals progressively greater than 1 (Fig. 3). The break-even points became smaller compared to the situation above with lower levels of air pollution exposure, being roughly closed to or even below 40 MET-h/d as the air pollution level increased. Specifically, the break-even points were 24 MET-h/d, 40 MET-h/d and 61 MET-h/d when PM_10_ was 120 µg/m^3^, PM_2.5_ was 70 µg/m^3^, and PM_1_ was 42 µg/m^3^, respectively.

Furthermore, the results of sensitivity analysis were similar to those of the primary analysis, showing that with high levels of air pollution exposure, the health benefits of PA were more significantly influenced by the exposure to PMs than with lower levels of air pollution exposure. (Figs. S3, S4 and S5).

## Discussion

### Main findings

To our knowledge, this is the first study to investigate the joint effects of air pollution and physical activity on type 2 diabetes in older adults exposed to widely varying levels of air pollution exposure. Based on the pattern shown in the results, this study indicated that with PM_10_ < 92 µg/m^3^, PM_2.5_ < 61 µg/m^3^, and PM_1_ < 36 µg/m^3^, the benefit effects of PA on type 2 diabetes was greater than the harmful effects due to PMs especially when PA levels were roughly blow 80 MET-h/d. However, with extreme high levels of PMs (PM_10_ ≥ 92 µg/m3, PM_2.5_ ≥ 61 µg/m3, and PM_1_ ≥ 36 µg/m3), the potential detrimental effects due to augmented exposure to air pollution during PA could outweigh the protective effects of PA, especially when PA levels were roughly above 40 MET-h/d.

### Potential mechanism

Exercise is known to reduce the risk of type 2 diabetes, and daily moderate- or high-intensity exercise is likely optimal to enhance insulin activity [55]. The beneficial association between PA and type 2 diabetes has been well established, and PA promotion has been recommended by the WHO for diabetes prevention. Conversely, insulin resistance has been considered as a potential mechanism for the harmful health effects of PM on type 2 diabetes. Apart from the experimental studies which suggested insulin resistance among mice and rats [56, 57], human epidemiological studies have also demonstrated insulin resistance after air pollution exposure [58, 59]. Besides, air pollution has also been shown to cause subclinical inflammation [60]. Therefore, engaging in PA in a polluted atmosphere might have detrimental effects on health due to the increased inhalation of air pollutants in spite of the health benefits of PA.

### Comparison with previous studies

So far, the trade-off between the potential harmful effects caused by augmented exposure to air pollution during PA and the health benefits of increased PA remains unclear. Some studies has revealed that there was no significant interaction effects between air pollution and PA on hypertension [20], lung function/respiratory diseases [21], myocardial infarction, mortality [28], and type 2 diabetes [29]. They also have shown that the health benefits of physical activity are larger than the risk from an increased inhaled dose of fine particles during active commuting [22, 61].

However, those studies were conducted in developed countries or regions, with low or moderate air pollution exposure settings [27], such as the mean (SD) of overall PM_10_ exposure was 50 (5.69) µg/m^3^ [29] or PM_2.5_ exposure was 26.1 (7.3) µg/m^3^ [20], in which air pollution levels was much lower than our study settings or other developing countries. WHO proposed an Air Quality Guidelines (AQG) and interim targets for PMs, in which the IT-1 target for PM_10_ and PM_2.5_ (70 µg/m^3^ and 35 µg/m^3^, respectively) levels are associated with an approximately 22% and 24% higher long-term mortality risk relative to the AQG level (15 µg/m^3^ and 5 µg/m^3^ for PM_10_ and PM_2.5_, respectively) [62]. We found that nearly 43.8% participants were living in a high exposure environment, which did not meet the WHO guidelines. So conclusions of those previous study could not be extrapolated to high-exposure air pollution settings, and our study did show some different findings.

Our study found that the PA health benefits could be considered to outweigh the harm caused by air pollution except extreme air pollution concentrations, which was similar to another previous studies [24, 63]. Specifically, one study showed that in areas with PM_2.5_ concentrations of 66 µg/m^3^, the tipping points, beyond which additional PA will not lead to more health benefits, were 1 h per day for cycling, and 6.25 h per day for walking [63]. At a similar PM_2.5_ concentrations, i.e. 65 µg/m^3^, our results showed a tipping point of about 21 MET-h/d, implying about 6.36 h per day for walking (Fig. 3B). Notably, studies investigating the joint effects of PA and air pollution on diabetes were indeed scarce, let alone examining the dose–response relationship in a more convincing settings. More research on the combined effects of PMs and PA on diabetes is urgently needed.

In addition, WHO recommended older adults should do at least 150–300 min of moderate-intensity aerobic PA per week, which would mean 15–30 MET-h/week if the moderate-intensity PA was set at 6 MET [64]. Although the mean level of PA in this study, i.e. 24.93 MET-h/d, is much higher than WHO guidelines, it is similar with other Chinese studies [43, 65–67]. For example, one study based on the China Kadoorie Biobank (CKB) cohort found that the mean level of PA was 22 MET-h/d [43]. This study also found that farmers accounted for about 44% of the participants, which had a higher mean level of PA than non-farmers (36.39 MET-h/d *vs.* 15.87 MET-h/d). Therefore, more research is needed to investigate the distribution of PA in China and to explore whether the WHO guidelines are appropriate for the Chinese context, so as to derive suitable PA guidelines for China.

### Calculation methods for the joints effects

Causal inference based on the IPTW has been widely popular and applied in observational studies, but there is a lack of IPTW studies on the joint or interaction effects of two continuous variables. This study proposed a bi-dimensional GPS, and then applied the IPTW method to investigate the joint effects of two continues exposure on health, which is a very common and inevitable situation in real life. Although some assumptions of causal inference are untestable, our study showed that the findings were reliable and robust by the exclusion of participants with self-reported disease.

### Strengths and limitations

This study has several important strengths. First, most studies have been conducted in North America and European countries with good air conditions [21, 22, 28], but this study targeted a population with a relatively high level of air pollution exposure. The wide range of air pollution could provide valid evidence not only for low/moderate polluted areas, but also for severely polluted regions. Second, it is clinically very important to determine the optimal patterns of PA behaviours according to air pollution levels [24]. This study filled an evidence gap for adjusting PA behaviour to air pollution levels to prevent diabetes. Third, the large number of participants provided sufficient power to investigate the joint associations of PA and ambient PM_10_, PM_2.5_, or PM_1_ exposure and to obtain stable and precise estimates. Finally, a spatiotemporal model was used to estimate the concentrations of ambient PM_10_, PM_2.5_ and PM_1_ at a high resolution (1 × 1 km^2^), and indoor air pollution was included in our analysis.

This study also has a few limitations. First, we did not distinguish between indoor and outdoor PA. Thus, we could not exclusively examine the joint associations of outdoor PA and ambient PM_10_, PM_2.5_, and PM_1_ exposure with diabetes. Second, although the cross-sectional study design was a limitation for our study, we tried to use a study design to minimize the problem of inversion of cause and effect, such as excluding participants diagnosed with type 2 diabetes 3 years earlier and including only participants with at least 3 years of stable residence. Finally, we adopted a questionnaire to collect information related to exposure, such as smoking, drinking, PA, indoor pollution situation and dietary habits; thus, recall bias cannot be avoided, and misclassification might have occurred.

## Conclusions

In conclusion, these findings suggest that for the prevention of type 2 diabetes in older adults, the health benefits of PA could outweigh the harms caused by air pollution, except in extreme air pollution situations, and that PA levels should not exceed 40 MET-h/d when the air quality of residence is severe. More robust research on the dose–response relationship is warranted to validate our findings with a cohort study design in future research.

## Supplementary Information

**Additional file 1.**

## Data Availability

The CMEC datasets generated during and/or analysed during the current study are available from the corresponding author on reasonable request. The air pollution datasets generated during and/or analysed during the current study are available from the co-author (Jing Wei, Ph.D., email: weijing_rs@163.com) on reasonable request, https://weijing-rs.github.io/product.html.

## Abbreviations

PM_10_: Particulate matter with an aerodynamic diameter of 10 µm or less
PM_2.5_: Particulate matter with an aerodynamic diameter of 2.5 µm or less
PM_1_: Particulate matter with an aerodynamic diameter of 1 µm or less
PA: Physical Activity; METs-h/d: hours of metabolic equivalent tasks per day
OR: Odds ratio
SD: Standard deviation
GPS: Generalized propensity score
AC: Absolute correlation
