# Supplementary material for: The joint effects of physical activity and air pollution on type 2 diabetes in older adults
Source: BMC Geriatr. 2022 Jun 1;22:472. doi: 10.1186/s12877-022-03139-8 (PMC9158242; doi:10.1186/s12877-022-03139-8)
Supplement: Supplementary file 1 — Additional file 1. [file 12877_2022_3139_MOESM1_ESM.docx]

Supplementary Materials for

**The joint effects of physical activity and air pollution on type 2 diabetes in older adults**

Linjun Ao^1†^, Junmin Zhou^1†^, Mingming Han^2^, Hong Li^3^, Yajie Li^4^, Yongyue Pan^5^, Jiayi Chen^1^, Xiaofen Xie^1^, Ye Jiang^1^, Jing Wei^6^, Gongbo Chen^7^, Shanshan Li^8^, Yuming Guo^8^, Feng Hong^9^, Zhifeng Li^10^, Xiong Xiao^1*^, Xing Zhao^1^

^*^ Corresponding author. xiaoxiong.scu@scu.edu.cn

**This file includes:**

Supplementary Text S1

Figs. S1 to S5

Tables S1

**Supplementary Text S1**

**Proof**

$$f_{T,V}\left\{ \left( t,v \right) | r\left( t,v,X \right),Y\left( t,v \right) \right\}=f_{T,V}\left\{ \left( t,v \right) | r\left( t,v,X \right) \right\}$$

$$f_{T,V}\left\{ \left( t,v \right) | r\left( t,v,X \right),Y\left( t,v \right) \right\}$$

=$\int f_{T,V}\left\{ \left( t,v \right) | x,r\left( t,v,X \right),Y\left( t,v \right) \right\}dF_{X}\left( x | r\left( t,v,X \right),Y\left( t,v \right) \right)$

=$\int f_{T,V}\left\{ \left( t,v \right) | x \right\}dF_{X}\left( x | r\left( t,v,X \right),Y\left( t,v \right) \right)$

=$\int r\left( t,v,x \right)dF_{X}\left( x | r\left( t,v,X \right),Y\left( t,v \right) \right)$

=$r\left( t,v,X \right)$

$$f_{T,V}\left\{ \left( t,v \right) | r\left( t,v,X \right) \right\}$$

=$\int f_{T,V}\left\{ \left( t,v \right) | x,r\left( t,v,X \right) \right\}dF_{X}\left( x | r\left( t,v,X \right) \right)$

=$\int f_{T,V}\left\{ \left( t,v \right) | x \right\}dF_{X}\left( x | r\left( t,v,X \right) \right)$

**=**$\int r\left( t,v,x \right)dF_{X}\left( x | r\left( t,v,X \right) \right)$

=$r\left( t,v,X \right)$


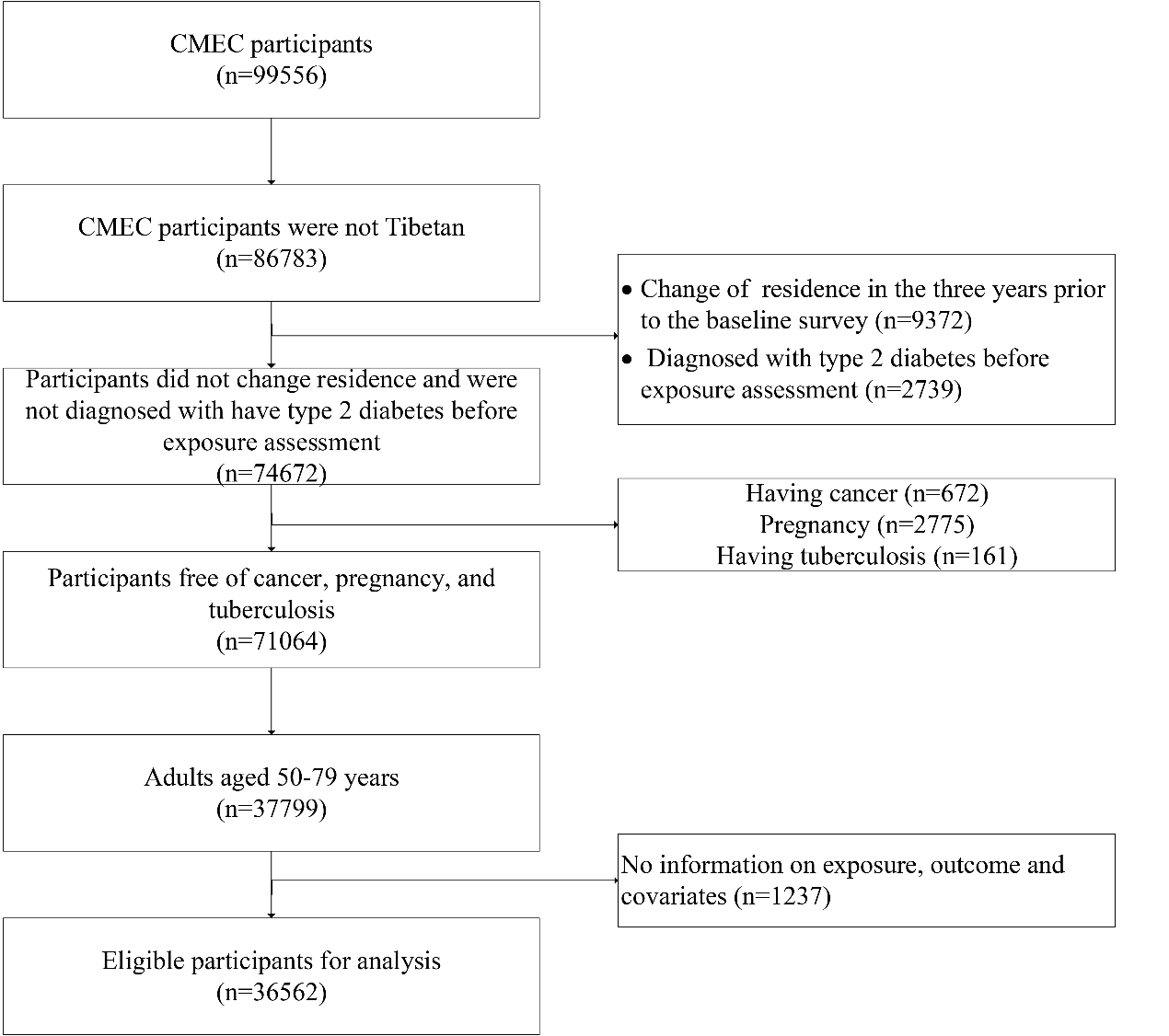


**Figure S1. Flow diagram of participants’ enrolment**

**
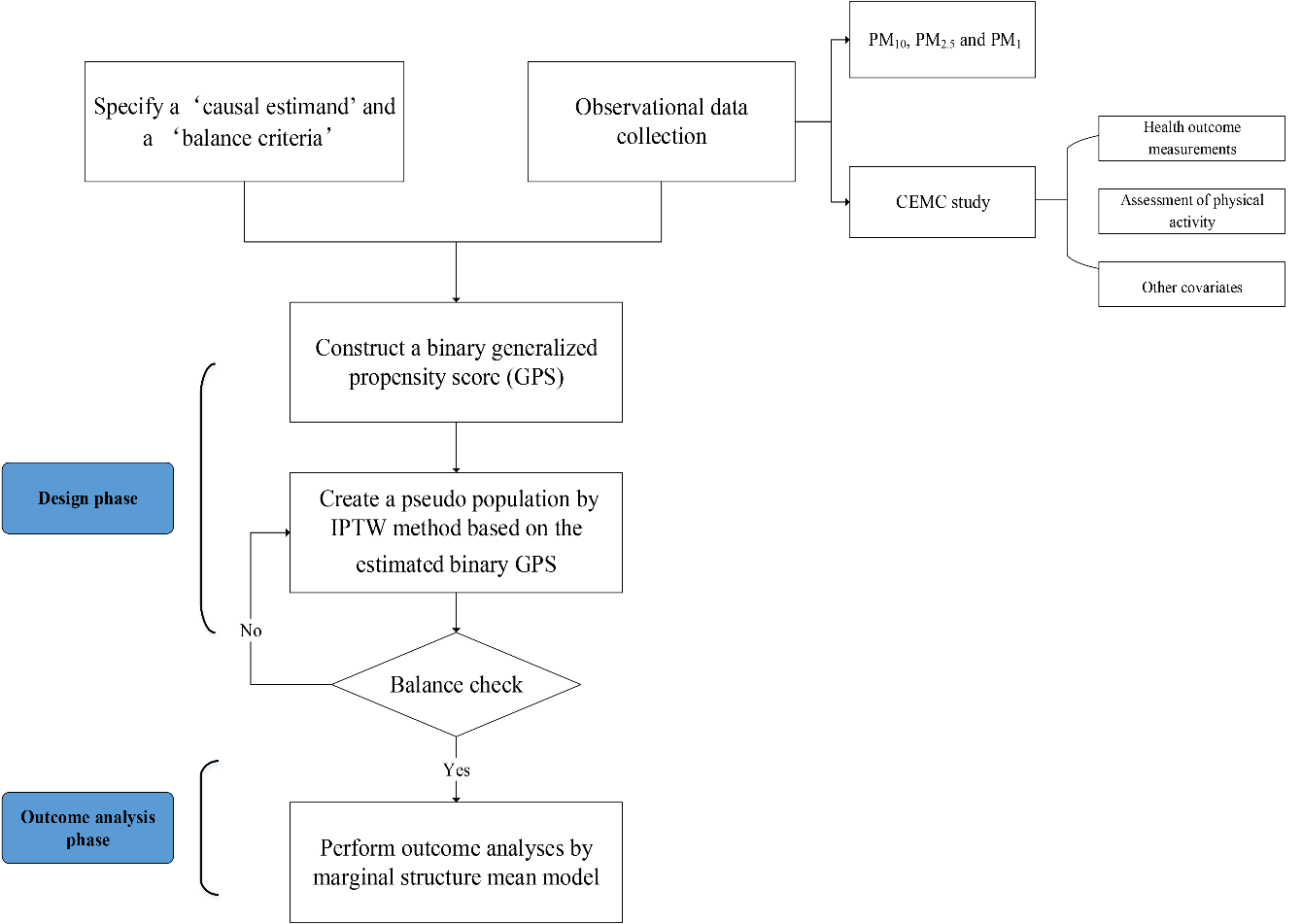
**

**Figure S2: Causal Inference Workflow.** A workflow for causal inference approaches using the estimated bi-dimensional GPS to design and analyze observational data. The design and analysis phases are kept separate, and the technical details about each phase are discussed in Methods section.

**Table S1. The characteristics of the study participants with different levels of physical activity.**

|  | **[0, 9.80]** | **(9.80, 20.17]** | **(20.17, 36.66]** | **(36.66, 139.49]** | ***P*** |
| --- | --- | --- | --- | --- | --- |
| **n** | 9146 | 9135 | 9140 | 9141 |  |
| **Age, mean (SD)** | 63.84 (7.91) | 61.15 (7.50) | 59.18 (6.82) | 58.00 (6.33) | <0.001 |
| **Type 2 diabetes, n (%) : yes** | 1222 (13.4) | 975 (10.7) | 932 (10.2) | 849 ( 9.3) | <0.001 |
| **PM_10_(µg/m^3^) , mean (SD)** | 76.31 (24.00) | 73.95 (23.89) | 67.36 (22.20) | 62.38 (20.41) | <0.001 |
| **PM_2.5_(µg/m^3^) , mean (SD)** | 44.76 (15.97) | 43.12 (16.00) | 38.67 (14.92) | 35.25 (13.82) | <0.001 |
| **PM_1_(µg/m^3^) , mean (SD)** | 29.46 (6.69) | 28.76 (6.64) | 26.85 (6.15) | 25.41 (5.71) | <0.001 |

The intervals [0, 9.80], (9.80, 20.17], (20.17, 36.66], (36.66, 139.49] are based on quartiles of physical activity (MET-h/d).


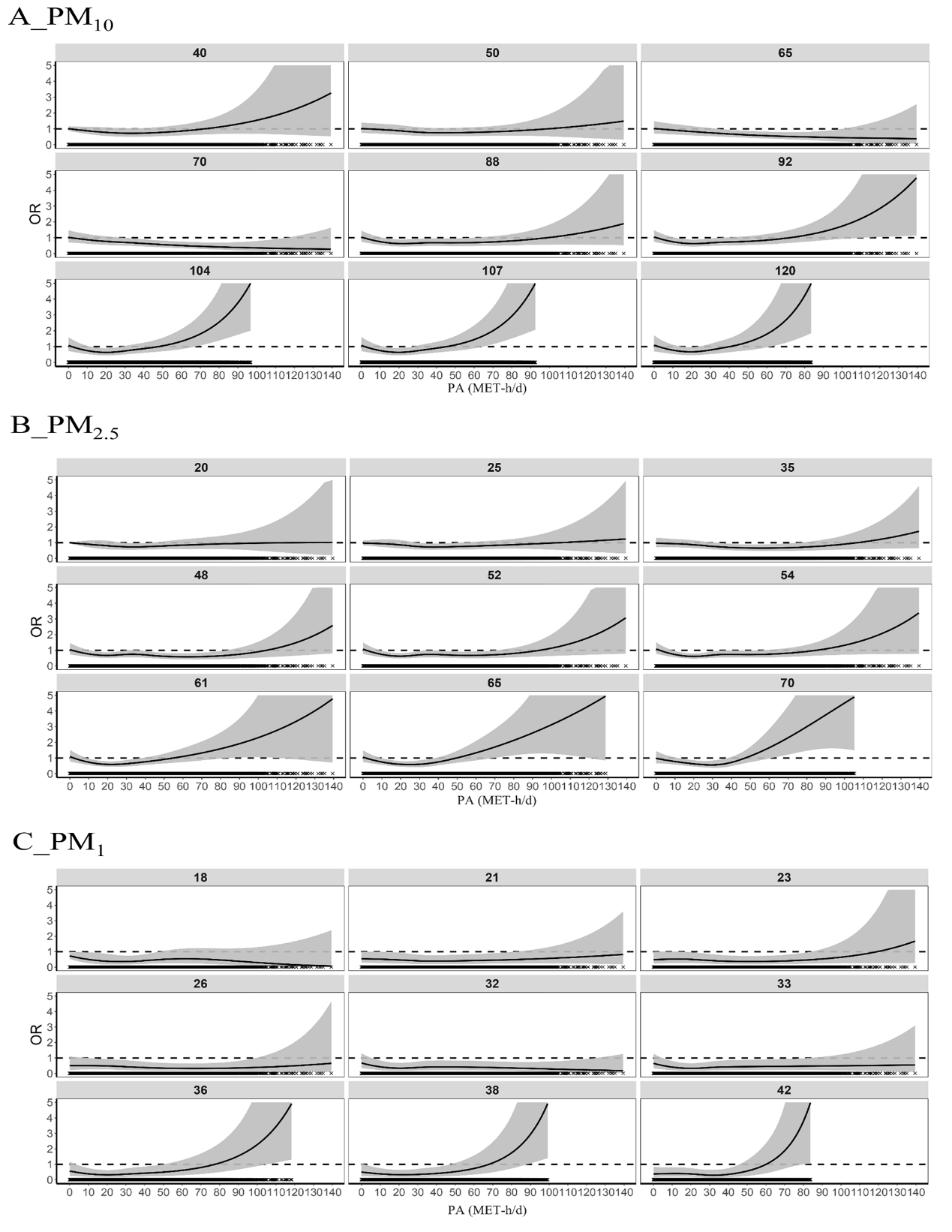


**Figure S3. The exposure-response relationship between PA and type 2 diabetes at different levels of 3-year average air pollution for older adults without taking any antidiabetic medication.** The nine values in subplots A, B and C represented the different pollution concentrations (µg/m^3^) of PM_10_, PM_2.5_ and PM_1_ respectively. The OR limit is set to 5, and the grey shaded area indicated the confidence interval (95% CI). Covariates mentioned in the Methods section were integrated by the bi-dimensional GPS, which was combined in the outcome model.

**
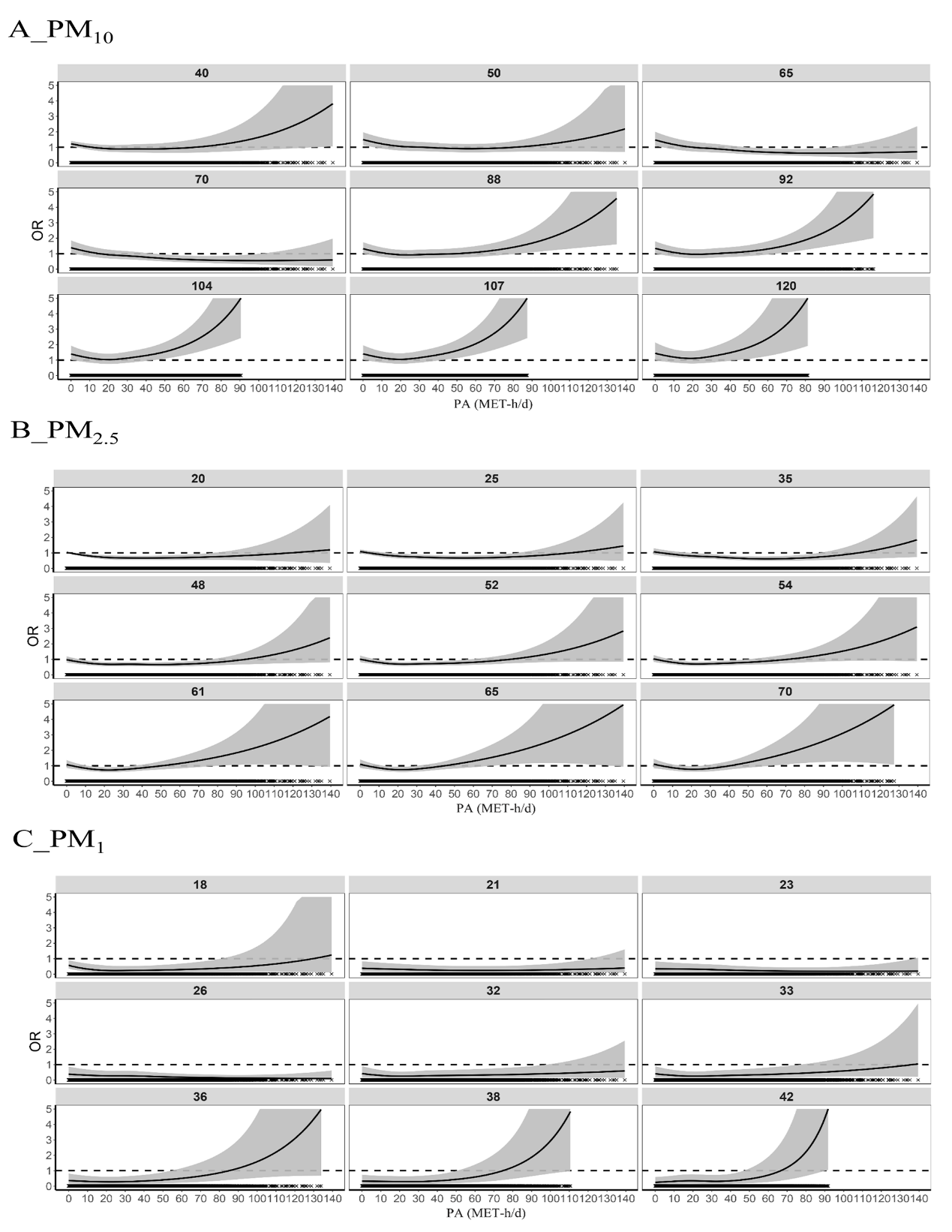
**

**Figure S4. The exposure-response relationship between PA and type 2 diabetes at different levels of 2-year average air pollution for older adults.** The nine values in subplots A, B and C represented the different pollution concentrations (µg/m^3^) of PM_10_, PM_2.5_ and PM_1_ respectively. The OR limit is set to 5, and the grey shaded area indicated the confidence interval (95% CI). Covariates mentioned in the Methods section were integrated by the bi-dimensional GPS, which was combined in the outcome model.

**
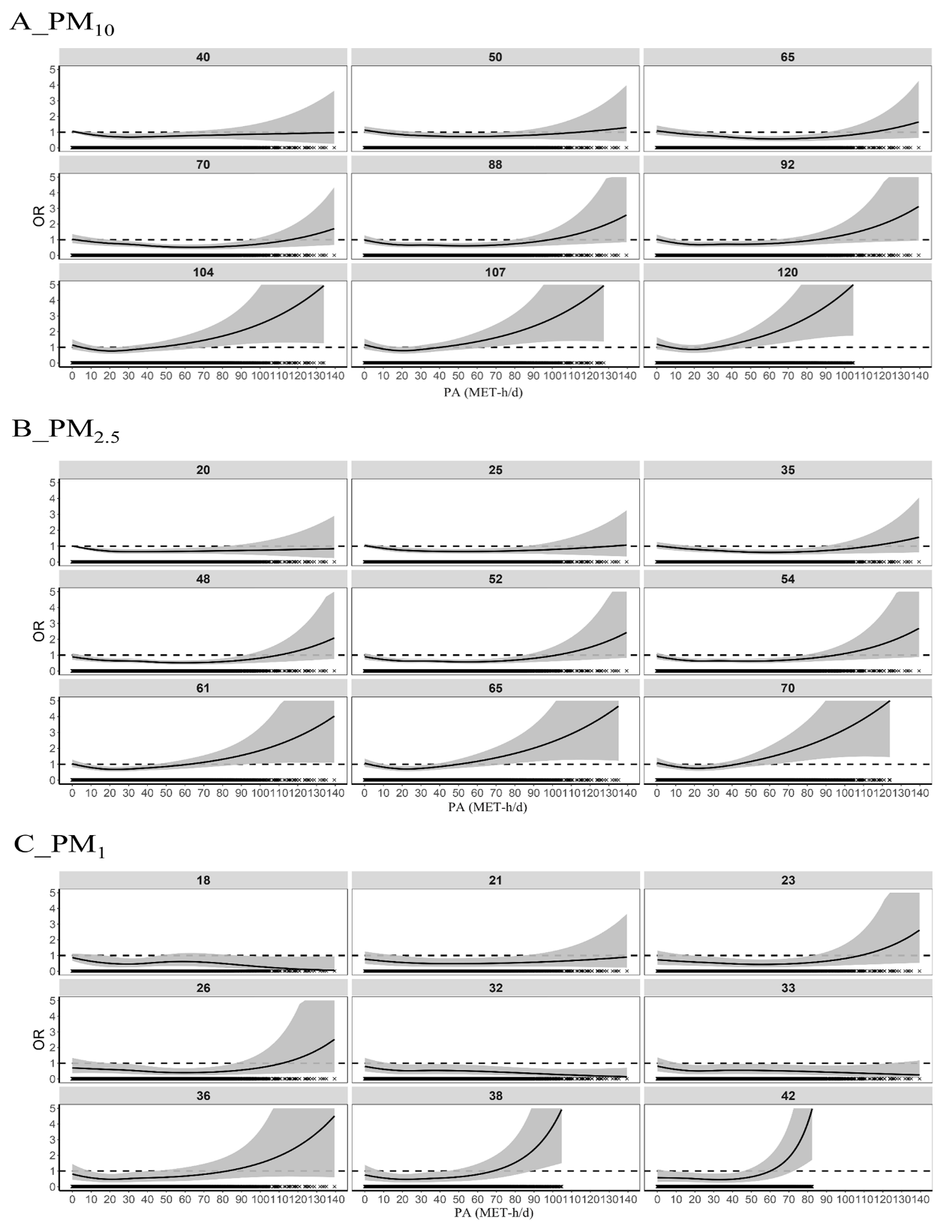
**

**Figure S5. The exposure-response relationship between PA and type 2 diabetes at different levels of 4-year average air pollution for older adults.** The nine values in subplots A, B and C represented the different pollution concentrations (µg/m^3^) of PM_10_, PM_2.5_ and PM_1_ respectively. The OR limit is set to 5, and the grey shaded area indicated the confidence interval (95% CI). Covariates mentioned in the Methods section were integrated by the bi-dimensional GPS, which was combined in the outcome model.
